# The use of generic versus brand names in (clinical) pharmacology education across Europe: a cross-sectional survey

**DOI:** 10.1007/s00228-026-04005-x

**Published:** 2026-02-10

**Authors:** Veronika Slezáková, Jitka Rychlíčková, Yoann Cazaubon, Erik M. Donker, Ellen van Leeuwen, Robert Likic, Dana Mazánková, Joost D. Piët, Fabrizio De Ponti, Walter Raasch, Floor van Rosse, Emilio J. Sanz, Markus Schwaninger, Michiel A. van Agtmael, Jelle Tichelaar

**Affiliations:** 1 Department of Clinical Pharmacy and Drug Information Centre, Institutional Pharmacy, Institute for Clinical and Experimental Medicine IKEM, Prague, Czech Republic; 2 Department of Applied Pharmacy, Faculty of Pharmacy, Masaryk University, Brno, Czech Republic; 3 Department of Pharmacology, Faculty of Medicine, Masaryk University, Kamenice 753/5, Brno, 625 00 Czech Republic; 4 St. Anne’s University Hospital, Pekarska, 664/53, Brno, 602 00 Czech Republic; 5 Department of Pharmacology, Montpellier University Hospital, 34090 Montpellier, France; 6 Pathogenesis and Control of Chronic and Emerging Infections (PCCEI), Univ Montpellier, INSERM, Univ Antilles, 34090 Montpellier, France; 7 Department of Internal Medicine, Unit Pharmacotherapy, Amsterdam UMC, Vrije Universiteit, De Boelelaan 1117, Amsterdam, 1081 HV The Netherlands; 8 Research and Expertise Centre in Pharmacotherapy Education (RECIPE), De Boelelaan 1117, Amsterdam, 1081 HV The Netherlands; 9 Department of Fundamental and Applied Medical Sciences, Unit of Clinical Pharmacology, Ghent University, C. Heymanslaan 10, Ghent, 9000 Belgium; 10 Unit of Clinical Pharmacology, Department of Internal Medicine, University Hospital Centre Zagreb and University of Zagreb School of Medicine, 12 Kišpatićeva St, Zagreb, 10 000 Croatia; 11 Department of Medical and Surgical Sciences, Pharmacology Unit, Alma Mater Studiorum, University of Bologna, Via Irnerio 48, Bologna, 40126 Italy; 12 Institute of Experimental and Clinical Pharmacology and Toxicology, University of Lübeck, Lübeck, Germany; 13 Erasmus MC, Department of Hospital Pharmacy, University Medical Center Rotterdam, Rotterdam, The Netherlands; 14 School of Health Science, Universidad de La Laguna and Hospital Universitario de Canarias (SCS), Santa Cruz de Tenerife, Calle Padre Herrera, S/N, La Laguna Tenerife, 38200 Spain; 15 Interprofessional Collaboration and Medication Safety, Faculty of Health, Sports and Social Work, InHolland University of Applied Sciences, Pina Bauschplein 4, 1095PN, Amsterdam, The Netherlands

**Keywords:** Pharmacology, Clinical pharmacology, Education, Brand names, Generic names

## Abstract

**Purpose:**

In pharmacology and clinical pharmacology (P&CP) teaching, using generic names of medicines contributes to global comprehensibility. However, brand names are often used in routine clinical practice, which may pose a challenge in the educational setting. This study aimed to explore P&CP teachers’ use of and attitudes towards generic and brand names in undergraduate education, as well as national prescription regulations.

**Methods:**

A survey was conducted with P&CP teachers from 38 European countries. An electronic questionnaire was used to collect data on the participants’ profiles, the nomenclature used and preferred in undergraduate education, and prescribing practices. The latter were then verified with national regulatory authorities.

**Results:**

Sixty-one teachers from 23 countries participated in the study, the majority being physicians (*n* = 47). In total, 13 (21%) teachers use only generic names in their teaching, while 46 (75%) use both generic and brand names. Thirty-four (56%) teachers preferred listing brand names in case-based teaching, as this reflects real life. Conversely, 37 (61%) teachers stated that students should not be exposed to brand names before graduation. Generic prescribing is either allowed or mandatory in all participating countries except two, where brand-name prescribing is mandatory.

**Conclusion:**

This study shows that generic names must remain fundamental in P&CP education, but brand names also play a role, especially in case-based education, reflecting real-life practice. Potential implications of generic and brand prescribing for internationalisation in education could be worth further attention.

**Supplementary Information:**

The online version contains supplementary material available at 10.1007/s00228-026-04005-x.

## Introduction

An integral component of pharmacology and clinical pharmacology education is understanding the fundamental principles of drug mechanisms of action and acquiring knowledge of their profiles. In order to harmonise pharmacology and clinical pharmacology education across Europe, Donker et al. focused on identifying key drugs that junior doctors working in Europe should be able to prescribe. A list comprising 98 items was published, with the generic nomenclature employed [1]. Generic names facilitate global comprehensibility, uniformity, and uniqueness, thereby ensuring unambiguous identification, clear communication, and contributing to preventing medication errors. Nevertheless, brand names may predominate in routine clinical practice and prescribing [2, 3]. In such circumstances, graduates must demonstrate a capacity for expeditious adaptation to real-life drug terminology, with brand names often lacking any logical connection to generic names taught at the university.

The gap in knowledge of generic and brand names of drugs was well illustrated by Davis et al. in their prospective study, where participants were tasked with identifying the generic names of 10 common, 5 high-risk, and 5 uncommon brand names. Only 36% of participants achieved an acceptable result. The least correct answers were provided by medical students, followed by interns/resident medical officers and consultants; fellows achieved the highest rate. These results clearly demonstrate that the knowledge of brand names is not taught at universities, but rather learnt “on the job” [3]. Brand names thus pose a challenge in the educational setting. Moreover, the medical teachers’ approach plays an important role. Ryskina et al. provided an insight into the supervisory impact on residents’ prescribing practice of brand vs. generic name statins. The higher the attending’s brand-name prescribing rate, the higher the odds of a resident prescribing a brand-name statin [4]. The significant role of the medical teacher was confirmed by Tichelaar et al. in their analysis of what final-year medical students base their drug choice on, as was also the case with Hartjes et al., who underscore the role of reasoning in the therapy choice, which the supervisor should provide when teaching students and residents in order to avoid ingrained pattern adoption [5, 6]. It can be expected that this issue affects not only medical students, but also pharmacy, nursing or any other students of healthcare majors. When considering these aspects in the context of undergraduate education, educators should recognise their role and the potential, albeit unintentional, promotion of particular products or brand-name prescriptions in general. In addition, they should address the potential health economic implications of prescribing practices, as the choice between generic and brand-name drugs can significantly influence healthcare costs at both individual and system levels.

In the light of the above, the objective of this study was to ascertain the extent to which brand names are utilised in undergraduate education and to explore the attitudes of pharmacology and clinical pharmacology teachers towards this issue in the context of established rules and real-life practice.

## Methods

This cross-sectional survey involved pharmacology and clinical pharmacology teachers across 38 European countries. The questionnaire was distributed both physically (at the ERASMUS+ Clinical Pharmacology and Therapeutics Teach the Teacher (CP4T) project event – Teach the Teacher course, Amsterdam, May 13–14, 2025) and electronically through Google Forms (368 university teachers of pharmacology and clinical pharmacology in the Network of Teachers in Pharmacotherapy (NOTIP) covering all EU countries, the United Kingdom, Norway, and Serbia, June 3–July 8, 2025, including a reminder on June 24, 2025). In addition to the requirement to participate in pharmacology and clinical pharmacology teaching, as evidenced by participation in the NOTIP or ERASMUS+ CP4T project (the CP4T project helps to support and train educators in the field of clinical pharmacology and therapeutics with the aim of harmonising and modernising the teaching of drug prescribing across Europe), we did not apply any other criteria for inclusion or exclusion [7]. 

The web-based questionnaire was organised into four sections: consent to participate (each participant was asked for the consent to participate), participant profile, personal opinion on using generic versus brand names in pharmacology and clinical pharmacology courses, and local prescribing practices. Prior to the dissemination, the questionnaire was validated (internally discussed in terms of clarity, relevance) and piloted (comprehensibility, technical functionality) by the CP4T project partners. The full version is available in the Supplementary materials. “Generic names” refer to the globally recognised public names (listed as International Nonproprietary Names, INN). INN and generic names are used interchangeably in this paper. The questionnaire provided an example of “amlodipine”. The term “brand names” referred to trademarked names, and the questionnaire provided a randomly selected example of “Agen®” [8, 9]. Teacher-centred education in basic pharmacology and pharmacology of particular drug classes, providing theoretical knowledge, was regarded as “theoretical subjects”. In contrast, student-centred education, intended for students with basic knowledge of pharmacology, focused on medication review, case-report analysis, was considered “applied subjects” for the purpose of our questionnaire.

The questionnaire was fully anonymised. The Ethics Committee of the Faculty of Medicine at Masaryk University has confirmed that the survey does not require their approval and that the requirement for informed consent from the participants can be waived. The research carried here is in compliance with the Declaration of Helsinki principles. The survey did not obtain a clinical trial number as it was not applicable. The questionnaire was reported in accordance with the CROSS guidelines.

The collected answers were downloaded in MS Excel format for subsequent analysis. Data were analysed using descriptive statistics only given the exploratory nature of the study. Responses were summarised as absolute and relative frequencies for each category.

In addition to collecting data on prescribing practices through the questionnaire, we verified the basic rules and regulations governing prescribing in each participating country by contacting national regulatory authorities, and official website screening when necessary. Participant responses were compared against these verified sources, and any discrepancies were recorded as inconsistencies with the assumption that the regulatory information represented the correct standard. This verification was undertaken to ensure accuracy and minimize bias.

## Results

### Participants profile

Sixty-one participants from 23 European countries completed the questionnaire. An overview of their characteristics is provided in Table 1. Over three-quarters of the participants were physicians, the primary healthcare professionals permitted to prescribe drugs in all countries (see below). Simultaneously, more than half of the participants were engaged in both clinical practice and academia. All participants had more than 1 year of experience in their respective fields.

**Table 1:** Participants’ profile

**number of participants per country**	**country**
1	Croatia, Estonia, Ireland, Kosovo, Montenegro, Norway, Serbia, Slovakia, Slovenia, Sweden, Turkey
2	Bulgaria, Portugal, Romania, United Kingdom
3 and more	Czech Republic, France, Germany, Italy, Latvia, Netherlands, Poland, Spain
**Participant background**	**number of answers (%)**
Physicians	47 (77.1%)
pharmacists	11 (18.0%)
Other	3 (4.9%)
**main occupation**	**number of answers (%)**
academic	22 (36.1%)
clinical	5 (8.2%)
academic and clinical roughly the same	34 (55.7%)
**time spent in this position**	**number of answers (%)**
1–5 years	5 (8.2%)
5–10 years	4 (6.6%)
10–15 years	6 (9.8%)
15–20 years	8 (13.1%)
20 + years	38 (62.3%)

### Drug names in pharmacology and clinical pharmacology education

In pharmacology education, generic drug names were utilised predominantly (96.7%), with two-thirds of participants occasionally combining them with brand names (see Table 2). If participants reported differences in the use of drug names between theoretical and applied subjects, brand names were more frequently used in applied subjects. While in theoretical subjects, brand names were typically mentioned only for specific drug formulations or when substitution was not possible (e.g., specific drug release).

**Table 2:** Attitudes to drug names used and preferred in pharmacology and clinical pharmacology classes

Question		Answers	No of answers (%)
1	How are students at your university taught about drug names during pharmacology lessons?	INN only	13 (21.3)
		INN only, but brand names sometimes mentioned (always together with the INN)	41 (67.2)
		both INN and brand names	5 (8.2)
		brand names only	1 (1.6)
		don’t know	1 (1.6)
2	Is there a difference in using drug names between purely theoretical and applied subjects?	no, there is no difference, we teach theoretical and applied subjects the same way regarding drug names	28 (45.9)
		yes, there is a difference, theoretical subjects use INN, applied subjects brand names	21 (34.4)
		yes, there is a difference, theoretical subjects use brand names, applied subjects INN	0 (0.0)
		don’t know	12 (19.7)
3	Do you personally think students should be taught about brand names before graduating?	yes, I think students should be taught about brand names	14 (23.0)
		no, I think their first contact with brand names should be after graduation	37 (60.7)
		other (please elaborate)	10 (16.3)
4	Do you think students should come into contact with brand names at least during case studies focusing on medication review of patients from real life?	no, drugs should be listed by INN only	27 (44.3)
		yes, drugs should be listed by brand names to show what was really given to patient	34 (55.7)
5	Do you think there should be a difference in teaching drug names between medicine, nursing and pharmacy students?	no	56 (91.8)
		yes, medicine and nursing students should be taught more about brand names	0 (0.0)
		yes, pharmacy students should be taught more about brand names	5 (8.2)
6	If brand names were to be introduced into pharmacology subjects, how should be decided in which areas should be brand names taught?	theoretical subjects only, with focus on the most frequently prescribed drug groups	10 (16.4)
		theoretical subjects only, with as wide scope of drug groups as possible to provide comprehensive overview	0 (0.0)
		applied subjects only, with focus on the most frequently prescribed drug groups	15 (24.6)
		applied subjects only, with as wide scope of drug groups as possible to provide comprehensive overview	3 (4.9)
		both theoretical and applied subjects, with focus on the most frequently prescribed drug groups	14 (23.0)
		both theoretical and applied subjects, with as wide scope of drug groups as possible to provide comprehensive overview	7 (11.5)
		other (please elaborate)	12 (19.6)
7	Should there be placed the same expectations on domestic and international students regarding brand names or should they differ?	both domestic and international students should have the same knowledge about brand names in our country	32 (52.5)
		international students don’t need to know as much about brand names in our country as domestic students	20 (32.8)
		other (please elaborate)	9 (14.7)

A total of 60.7% of participants expressed a preference for students to encounter brand names after graduation. However, upon narrowing the question to applied subjects, such as medication reviews of real patients and case-based teaching, the ratio is inverted, with 55.7% of participants agreeing with providing students with brand names of medications used by patients before graduation. The hypothetical question regarding introducing brand names into pharmacology and clinical pharmacology teaching (question 6, Table 2) highlighted the variability of participants’ attitudes. However, there was a general consensus in favour of concentrating on the most frequently prescribed medications.

The expected benefits of introducing brand names were a better understanding of real-life situations, improved preparation for real practice, and augmented ability to use drug databases (32.8%). Furthermore, 10% of the participants underscored that brand names are often preferred in healthcare professionals’ communication. Another perspective was potentially improved communication with patients, who frequently know brand names alone. Other benefits mentioned were enhanced integration of theoretical and applied subjects and the potential for facilitating the memorisation of generics. For pharmacy students, recognising brand names might be a benefit for their future practice.

Still, more than one-third of participants (37.7%) did not perceive any benefits, and there were participants who did not respond to the question at all. The anticipated adverse outcomes were the bias instilled in the students and the promotion of a marketing holder (31.2%), both with long-term consequences. Other major concerns included the potential for an extra workload for students, the utilisation of brand names as a source of confusion, and the prioritisation of learning brand names over generic ones. The spectrum, variability, and lack of a worldwide harmonisation of brand names and dynamic changes in product availability in the market were emphasized too. Two participants warned that students might be familiar with brand names but not the active ingredients, which was closely connected to the dangers of incorrect and double prescriptions.

### Prescribing practices according to drug names allowed, used, preferred

In all countries, physicians were authorised to prescribe medications; in some countries, nurses, midwives, and pharmacists were also authorised to do so. In all the countries covered, prescribing using generic names was at least permitted, if not mandatory (see Table 3). An inconsistency between the participants’ answers and the information from the national competent authorities was identified in 24 participants from 14 countries. Most often, participants selected “INN mandatory, brand name allowed” instead of the more appropriate “both INN and brand name allowed, none mandatory”, and vice versa. The legislative framework governing prescribing practices remained largely unchanged over recent years. Only the Czech Republic, France, Latvia, and Portugal reported significant changes in the last five years (specifically shifting towards generic prescription being allowed or mandatory).

**Table 3:** Countries according to the type of prescription allowed

Type of prescription	Countries
both INN and brand name allowed, none mandatory	Bulgaria, Czech Republic, Germany, Ireland, Montenegro, Norway, Poland, Romania, Serbia, Slovenia, Turkey, the United Kingdom
INN mandatory, brand name allowed	Estonia, France, Italy, Latvia, Netherlands, Portugal, Slovakia, Spain
brand name mandatory, INN allowed	Croatia, Sweden
INN mandatory, brand name prohibited	Kosovo
brand name mandatory, INN prohibited	none

In most countries, the criteria applied to inpatient and outpatient prescribing were largely similar; if differences existed, generic prescribing was more prevalent in the inpatient setting and was coupled with positive lists of preferentially prescribed drugs. An overview of the generic prescribing and generic substitution aspects can be found in Table 4.

**Table 4:** Aspects of generic prescribing, generic substitution

Question		Answers	No of answers (%)
1	If you could choose, how would you prefer to prescribe regardless of your country’s current situation and your working position?	INN prescription	35 (57.4)
		brand name prescription	4 (6.6)
		both brand name and INN prescription	22 (36.0)
2	Who can prescribe in your country? (multiple choice)	doctors	61 (100.0)
		pharmacists	6 (9.8)
		nurses	19 (31.1)
		midwives	13 (21.3)
		don’t know	0 (0.0)
		other (please elaborate)	8 (13.1)
3	Can the dispensing pharmacist access patient’s drug history?	yes	30 (49.2)
		no	17 (27.9)
		don’t know	4 (6.6)
		other (please elaborate)	10 (16.3)
4	Is generic substitution allowed in your country?	yes	58 (95.1)
		no	1 (1.6)
		don’t know	2 (3.3)
5	Is it possible to substitute biologic drugs for biosimilars in your country?	yes	44 (72.1)
		no	7 (11.5)
		don’t know	10 (16.4)
6	Who can make the substitution of a biologic drug for a biosimilar?	substitution of biologic drugs not possible	1 (1.6)
		prescribing doctor only	29 (47.5)
		pharmacist with an approval of the prescribing doctor	6 (9.8)
		pharmacist, an approval of the prescribing doctor not necessary	9 (14.8)
		don’t know	12 (19.7)
		other (please elaborate)	4 (6.6)
7	Is there a difference in prescribing between ambulatory practice and hospitals/inpatient prescribing in terms of INN and brand names? (if yes, please select “Other” option and provide us with some details)	no	38 (62.3)
		don’t know	10 (16.4)
		other (please elaborate)	13 (21.3)

The regulatory framework was consistent with the preferences of the participants, who favoured generic prescribing (only generic or both generic and brand name prescribing to be allowed). However, the reality described by participants contrasted sharply with this; namely, the widespread use of brand names in routine clinical practice prescribing, even in countries with mandatory generic prescription. The most frequently reported reasons for prescribing brand name products were the narrow therapeutic window of the prescribed drug, patient intolerance to generics, and prescriptions for elderly patients with multiple comorbidities and prevalent cognitive impairments.

## Discussion

Pharmacology and clinical pharmacology are integral and relevant parts of the education of all healthcare professionals. A notable challenge in this area is the drug nomenclature employed. The findings of this study indicate that the education in Europe relies primarily on generic names, with only occasional inclusion of brand names particularly in applied subjects. In contrast, participants reported that routine clinical practice tends to favour brand names. This discrepancy, consistent with previously published data [2, 3], becomes especially evident during the transition from undergraduate education to clinical practice, where graduates are required to demonstrate prescribing skills upon entry, yet final-year students often lack confidence in their prescribing competencies [10, 11]. As the results of our prescribing practices mapping show, this gap may not only apply to medical students/physicians as generally authorized prescribers, but also to other healthcare professions authorized to prescribe in some countries. Participants in our study are highly aware of the discrepancy between the terminology used in education and clinical practice, as evidenced by their openness to incorporating brand names into teaching, or rather, their change in attitude toward their inclusion after focusing on case-based teaching. We are not aware of any other similar study that would allow these attitudes to be further analysed or compared.

To address this imbalance between preferentially generic names-based undergraduate education and dominantly brand names-based clinical practice, two approaches can be adopted to prevent medication errors stemming from the lack of knowledge of active ingredients in brand name drugs and increase prescribing safety: mandatory generic prescribing, including regulatory mechanisms to ensure its actual use, or adjustments in undergraduate education to prepare future healthcare workers for differences in communication they will face after graduation. This idea is also proposed by Davis et al. [3] The first approach is undoubtedly more rational and should be considered the ultimate goal, as the findings of this study demonstrate that generic prescribing is already at least possible in the majority of the countries reached. Time will likely play a role in implementing the mandatory generic prescribing. A more detailed analysis of the responses provided by participants from France and Latvia, two countries where there has been a shift towards generic prescribing, reveals a significantly more pronounced opposition to the use of brand names in education among participants from France than in Latvia, where four out of six participants provided benefits of brand name use in education. One potential explanation could be the five-year time-lag between introducing regulatory reforms on mandatory generic prescribing in France and Latvia. This longer period of adjustment may have led French educators to adopt a more consistent position against the inclusion of brand names in education. In contrast, Latvian educators are still adapting to the relatively recent regulatory change [2, 12]. In addition to habits, the inadequate preparedness of the system may also hinder the full implementation of generic prescribing. For instance, in the Czech Republic, while generic prescribing is permitted by law, software designed for outpatient prescriptions or in-hospital prescription rules frequently necessitate using a brand name [13]. Resolving these issues should be a priority.

In the meantime, however, it is worth considering whether to modify undergraduate education, especially in countries where brand names are still predominantly used in clinical practice. The results of this survey may provide a guidance to those involved in the education of future healthcare professionals. If brand names were to be introduced into education, they should remain optional and limited to the most commonly used drugs. Their inclusion may be more appropriate in applied subjects, such as medication reviews or case-based teaching, where pharmacotherapy is presented in the context of a specific patient. Another area might be products with unique formulations or pharmacokinetic properties. Knowledge of brand names should not be prioritised over generic names. In such circumstances, using brand names could provide better orientation. A disclaimer may serve to indicate the absence of an advertisement. Brand names can open discussion about the general principles of drug nomenclature, the practices of drug manufacturers (payments, gifts), and their possible economic impact [14–18]. Another safety issue is the “look-alike, sound-alike” products, particularly when brand names are not harmonised, and the international discrepancies of generic and brand prescribing further enhance their potential incorrect interpretation. In a world with constantly growing globalisation and cross-border collaborations in education, using brand names specific to just one country is a step backwards, which might hinder further attempts at harmonisation. Meanwhile, generic names are an unambiguous way to harmonise education across Europe.

## Strengths and limitations

To our best knowledge, this is the first survey mapping opinions on pharmacology and clinical pharmacology education in terms of drug names use and preferences, and in the context of the prescribing practices (who is prescribing, what is allowed and mandatory, and what is the reality), as it is the context that should help transform undergraduate education to prepare students appropriately for clinical practice. The strength of our study is the profile of the participants, who mostly have an insight into both education and clinical practice.

Despite all the best efforts, there are several limitations. The most important is the generalisability of the subjective responses in combination with a generally low number of participants, unevenly distributed across Europe. Additionally, participant backgrounds were not evenly represented, as the majority were physician, while other healthcare professionals were underrepresented. In case of multiple participants from one country, we could not distinguish whether they were affiliated with the same institution. A considerable proportion of participants did not correctly describe their local prescribing practices according to the rules established. To avoid any potential bias, the responses to hypothetical questions regarding prescribing were withdrawn from the final analysis.

## Conclusion

The findings of this survey indicate that most European teachers of pharmacology and clinical pharmacology have a clear preference for generic names not only in education, but also in all areas of healthcare. Despite that, brand names are deeply ingrained within numerous healthcare systems. While the pharmacology and clinical pharmacology education should remain consistent in its use of generic nomenclature, incorporating brand names in clearly defined circumstances may prove advantageous, at least temporarily, until generic prescribing becomes well-established. Emphasis should be placed on the ethical introduction of this practice into education, as teachers need to remain mindful of their influence on students, which could lead to a bias that one manufacturer might be superior to others. Brand names should not be mandatory knowledge if they are to be incorporated. Both generic and brand names should be listed, accompanied by a disclaimer and an articulation of the fundamental principles, benefits, and drawbacks of generic and brand prescribing.

## Supplementary Information

Below is the link to the electronic supplementary material.

Supplementary Material 1

## Data Availability

The data from this questionnaire are available upon request by contacting the corresponding author.
