# Supplementary material for: The use of generic versus brand names in (clinical) pharmacology education across Europe: a cross-sectional survey
Source: Eur J Clin Pharmacol. 2026 Feb 10;82(3):77. doi: 10.1007/s00228-026-04005-x (PMC12886207; doi:10.1007/s00228-026-04005-x)

SUPPLEMENTARY MATERIALS

The use of generic versus brand names in (clinical) pharmacology education across Europe

S1 Questionnaire regarding drug prescriptions across Europe

Dear participant,

please share with us information on which names of drugs you prefer in teaching students. INN (international non-proprietary names, e.g. amlodipine) only, both INN and brand names, or brand names only (e.g. Agen®). Does it depend on the way how drugs are prescribed in your country? Should the students meet the brand names too, or is it something irrational for you?

Please let us understand your local prescribing practices and see your opinion on what names of drugs are and should be used in pharmacology and prescribing education.

This questionnaire takes approximately 15 minutes to complete.

We would like to thank you in advance for your time and answers which will provide us with invaluable data.

This survey is conducted under the ERASMUS+ (project CP4T, 2022-1-NL01-KA220-HED-000088069)
INN – international non-proprietary names

PRESCRIBING SYSTEM OF YOUR COUNTRY

1. **How are drugs prescribed in your country?**
    - brand name prescription mandatory, INN prescription prohibited
    - brand name prescription mandatory, INN prescription allowed
    - both brand name and INN prescription allowed, none of them mandatory
    - INN prescription mandatory, brand name prescription allowed
    - INN prescription mandatory, brand name prescription prohibited
    - other (please elaborate)
    - don't know
2. **Has it always been like this or have there been any significant changes in prescribing methods during last 5 years?**
    - no changes
    - some changes (please elaborate)
    - other (please elaborate)
    - don't know
3. **If both brand name and INN prescription are possible in your country, do you know which one is more commonly used?**
    - brand name prescription
    - INN prescription
    - both prescriptions roughly the same
    - don't know
    - not applicable – brand name or INN not possible
4. **If INN prescription is mandatory in your country, do you know if prescribing doctors prefer to prescribe drug by INN only or by both INN and brand name at the same time?**
    - doctors prefer to prescribe by INN only
    - doctors prefer to prescribe by INN and brand name together
    - other (please elaborate)
    - don't know
    - not applicable – INN not possible

5. **If INN prescription is possible in your country, is it possible for the prescribing doctor to ensure that patient will be given a certain dosage form (tablets, syrup...) or is it completely up to the joint decision of a pharmacist and patient?**
    - prescribing doctor can mark preferred dosage form on a prescription
    - dosage form is decided by both pharmacist and patient upon dispensation of the prescription without input from the prescribing doctor
    - other (please elaborate)
    - don't know
    - not applicable – INN not possible
6. **If INN prescription is possible in your country, can the dispensing pharmacist access patient's drug history to ensure that patient receives the same brand of drug every time for their chronic conditions?**
    - yes
    - no
    - don't know
    - not applicable – INN not possible
7. **If INN prescription is mandatory in your country, are there any drugs which are exempted from this rule?**
    - no, all drugs must be prescribed by INN
    - yes, there are certain drugs/drug groups which have exemption (please elaborate which drugs/drug groups)
    - don't know
    - not applicable – INN not possible
8. **If you could choose how would you prefer to prescribe regardless of your country's current situation?**
    - brand name prescription
    - INN prescription
    - both brand name and INN prescription
9. **Optional – please elaborate your previous answer.**
    - *insert you answer*
10. **Who can prescribe in your country?**
    - doctors only
    - doctors and nurses
    - doctors, nurses and midwives
    - doctors, nurses and pharmacists
    - doctors and pharmacists
    - other (please elaborate)
    - don't know
11. **Is generic substitution allowed in your country?**
    - yes
    - no
    - don't know
12. **If yes, please elaborate which healthcare workers are allowed to make the substitution (pharmacists, nurses, etc.)**
    - *insert your answer*
13. **Is in your country possible substitution of biologic drugs for biosimilars?**
    - yes
    - no
    - don't know

14. **If you said yes in previous question, who can make the substitution of biologic drug for biosimilar?**
    - prescribing doctor only
    - pharmacist with a knowledge of prescribing doctor
    - pharmacist, knowledge of prescribing doctor not necessary
    - other (please elaborate)
    - don’t know
15. **Is there a difference in prescribing between ambulatory practice and hospitals?**
    - no
    - yes (please elaborate)
    - don’t know

PHARMACOLOGY EDUCATION SYSTEM IN YOUR COUNTRY

16. **How are students at your university taught about drug names during pharmacology lessons?**
    - INN only
    - both INN and brand names
    - brand names only
    - don’t know
17. **Is there a difference in teaching drug names between purely theoretical and applied subjects?**
    - no, there is no difference, we teach theoretical and applied subjects the same way regarding drug names
    - yes, there is a difference, theoretical subjects use INN, applied subjects brand names
    - yes, there is a difference, theoretical subjects use brand names, applied subjects INN
    - other (please elaborate)
    - don’t know
18. **Do you personally think students should be taught about brand names before graduating?**
    - yes, I think students should be taught about brand names
    - no, I think their first contact with brand names should be after graduation
    - other (please elaborate)
19. **If you said yes, do you think students should know the brand names and be able to use them during for example exams or should be brand names taught only as something extra, additional but optional knowledge?**
    - it should be mandatory for students to know and use brand names during their studies
    - it should be an optional knowledge, not something necessary to know
    - other (please elaborate)
20. **Do you think students should come into contact with brand names at least during case studies focusing on medication review of patients from real life?**
    - no, drugs should be listed by INN only
    - yes, drugs should be listed by brand names to show what was really given to patient
21. **Do you think there should be a difference in teaching drug names between medicine, nursing and pharmacy students?**
    - no
    - yes, medicine and nursing students should be taught more about brand names
    - yes, pharmacy students should be taught more about brand names
    - other (please elaborate)
22. **Please elaborate what kind of benefits do you personally think would introducing brand names into teaching bring**
    - *insert your answer*
23. **Please elaborate what kind of negatives do you personally think would introducing brand names into teaching bring**
    - *insert your answer*
24. **If brand names were to be introduced into pharmacology subjects, how should be decided in which areas should be brand names taught?**

- theoretical subjects only, with focus on the most frequently prescribed drug groups
- theoretical subjects only, with as wide scope of drug groups as possible to provide comprehensive overview
- applied subjects only, with focus on the most frequently prescribed drug groups
- applied subjects only, with as wide scope of drug groups as possible to provide comprehensive overview
- both theoretical and applied subjects, with focus on the most frequently prescribed drug groups
- both theoretical and applied subjects, with as wide scope of drug groups as possible to provide comprehensive overview
- other (please elaborate)

25. **Should there be placed the same expectations on domestic and international students regarding brand names or should they differ?**
    - both domestic and international students should have the same knowledge about brand names in our country
    - international students don't need to know as much about brand names in our country as domestic students
    - other (please elaborate)

DEMOGRAPHIC DATA

26. **What country are you from?**
    - *insert your answer*
27. **What is your background?**
    - physician
    - pharmacist
    - nurse
    - midwife
    - other (please elaborate)
28. **What is your current main occupation?**
    - academic
    - clinical
    - other (please elaborate)
29. **How long have you been working on this position?**
    - less than 1 year
    - 1-5 years
    - 5-10 years
    - 10-15 years
    - 15-20 years
    - 20+ years

S2 Leaflet used to promote the survey at the ERASMUS+ CP4T project Teach the Teacher course, Amsterdam, May 13-14, 2025

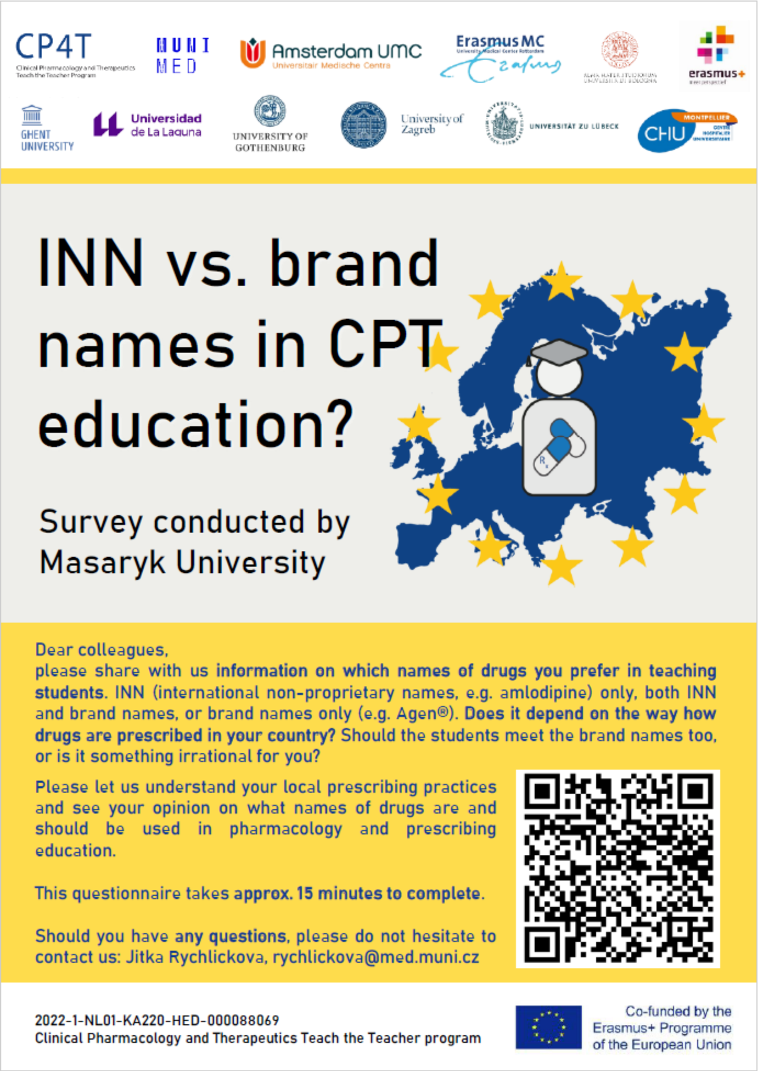

Supplement: Supplementary file 1 — Supplementary Material 1 [file 228_2026_4005_MOESM1_ESM.pdf]
